# Modeling the functions of condensin in chromosome shaping and segregation

**DOI:** 10.1371/journal.pcbi.1006152

**Published:** 2018-06-18

**Authors:** Yuji Sakai, Atsushi Mochizuki, Kazuhisa Kinoshita, Tatsuya Hirano, Masashi Tachikawa

**Affiliations:** 1 iTHES Research Group, RIKEN, Wako, Japan; 2 Theoretical Biology Laboratory, RIKEN, Wako, Japan; 3 Department of Biochemistry and Molecular Biology, Graduate School of Medicine, The University of Tokyo, Tokyo, Japan; 4 iTHEMS Program, RIKEN, Wako, Japan; 5 CREST, JST 4-1-8 Honcho, Kawaguchi, Japan; 6 Chromosome Dynamics Laboratory, RIKEN, Wako, Japan; Rutgers University, UNITED STATES

## Abstract

The mechanistic details underlying the assembly of rod-shaped chromosomes during mitosis and how they segregate from each other to act as individually mobile units remain largely unknown. Here, we construct a coarse-grained physical model of chromosomal DNA and condensins, a class of large protein complexes that plays key roles in these processes. We assume that condensins have two molecular activities: consecutive loop formation in DNA and inter-condensin attractions. Our simulation demonstrates that both of these activities and their balancing acts are essential for the efficient shaping and segregation of mitotic chromosomes. Our results also demonstrate that the shaping and segregation processes are strongly correlated, implying their mechanistic coupling during mitotic chromosome assembly. Our results highlight the functional importance of inter-condensin attractions in chromosome shaping and segregation.

## Author summary

Immediately before a cell divides, chromosomal DNA in a eukaryotic cell is packaged into a discrete set of rod-shaped chromosomes. This process, known as mitotic chromosome assembly or condensation, secures the faithful segregation of genetic information into daughter cells. Central to this mechanistically complex process is a class of protein complexes known as condensins. However, how condensins support the assembly and segregation of mitotic chromosomes at a mechanistic level remains elusive. Here we construct a coarse-grained physical model of chromosomal DNA fibers and condensin molecules, and study how condensins work in the mitotic chromosome assembly using computer simulations. Our results show that two activities of condensins, formation of consecutive loops in chromosomal DNA fibers and inter-condensin attractions, are necessary for both the shaping and segregation of mitotic chromosomes, and balancing acts of these activities help to coordinate the efficient progress of the processes. Importantly, chromosome shaping and segregation in our results are strongly correlated, implying that they are controlled by the same underlying mechanism mediated by condensins.

## Introduction

The assembly of rod-shaped chromosomes is one of the most dramatic events occurring during the eukaryotic cell cycle. Upon entry into mitosis, the mass of chromatin distributed within the interphase nucleus is converted into a discrete set of rod-shaped chromosomes. This process, commonly referred to as mitotic chromosome condensation, helps to relieve the entanglements created between duplicated sister chromatids and between different chromosomes, thereby ensuring the equal segregation of genetic information into daughter cells. Despite the long history of chromosome research, the mechanistic details of how such rod-shaped chromosomes might be assembled from long DNA molecules and a myriad of associated proteins remain a substantial mystery [1, 2].

One of the classical models in this field predicted that in a metaphase chromosome, the chromatin fiber is folded into a series of loop structures (chromatin loops) of about a few hundred kilobases, and the bases of the loops are anchored by a large proteinaceous structure, which is located at the axis of the chromosome referred to as the chromosome scaffold (the scaffold-loop model) [3]. Data from recent Hi-C analyses, an extension of chromosome conformation capture, have provided evidence that the formation of consecutive loops could indeed underlie the assembly of mitotic chromosomes [4]. The major constituents of the chromosome scaffold was shown to include subunits of large protein complexes, now known as condensins, that play a central role in mitotic chromosome assembly and architecture [5, 6]. A recent reconstitution assay using a limited number of purified protein components has substantiated the central importance of condensins (in particular, condensin I) in mitotic chromosome assembly [7].

These classical and emerging lines of evidence led us to predict that condensins might have at least two distinct molecular activities: chromatin loop formation and inter-condensin attractions. For the loop formation activity, a model called the loop extrusion model, in which a loop extrusion factor (predicted to be condensins) captures base point of loops and actively extrudes the loops, has been proposed recently and examined intensively [8–10]. An alternative model, random crosslinking of distal DNA segments by condensins, has also been considered [11]. Moreover, accumulating lines of evidence strongly suggest that protein-protein interactions are likely to play an important role in the action of condensins and other SMC protein complexes [12–17]. The postulated inter-condensin attractions would also confer mitotic chromosomes with the properties of rigidity and high elasticity [18, 19].

Although these observations and predictions can help to illuminate the potential mechanism of action of condensins, there remains a sizable gap in knowledge between understanding each elementary process and its consequent effect on chromosome assembly. We reasoned that molecular dynamics simulations of a coarse-grained polymer model that incorporates the postulated condensin activities could be a promising approach to begin to fill this gap [20]. From a physical point of view, it seems that loop formation and inter-condensin attractions would have opposite effects on chromosome shaping and segregation. On the one hand, the axial structure at the bases of consecutive loops would elongate a chromosome, whereas inter-condensin attractions would collapse the axis and make the chromosome spherical. On the other hand, loop formation would enhance segregation because the created loops would repulse each other, whereas inter-condensin attractions would repress segregation.

In the current study, we modeled the functions of condensin in mitotic chromosome assembly. We show that both loop formation and inter-condensin attractions are necessary for active mitotic chromosome assembly, and that balancing acts of the two activities help to coordinate the efficient shaping and segregation of mitotic chromosomes. Furthermore, we show that the shaping and segregation processes are strongly correlated.

## Results

### Coarse-grained polymer model

We consider a chromosome as a flexible polymer chain composed of spherical monomers with a few tens of nanometers in diameter, each corresponding to about ten nucleosomes (i.e., a few kilobases of DNA). The natural length of springs between monomers is set to be the same. We simulated chains of 5,000 monomers, corresponding to a few tens of megabases of DNA, which is close to the size of the shortest arm of human chromosomes. For this study, we rescaled the monomer diameter *σ* and the natural length of springs *d*_*B*_ to be one. The excluded volume interaction among monomers was modeled using the Lennard-Jones potential with a cut-off at a maximum energy *ϵ*_cut_ = 1000*k_B_T*, where *k*_*B*_ is the Boltzmann constant and *T* is the effective temperature. We modeled the springs between monomers without excluded volume (phantom springs). A phantom spring can pass through another chain, which is mediated by the strand-passing activity of topoisomerase II. Note that the actual frequency of the strand passage events is small due to the excluded volume of the monomers connected by the springs.

**Fig 1:**
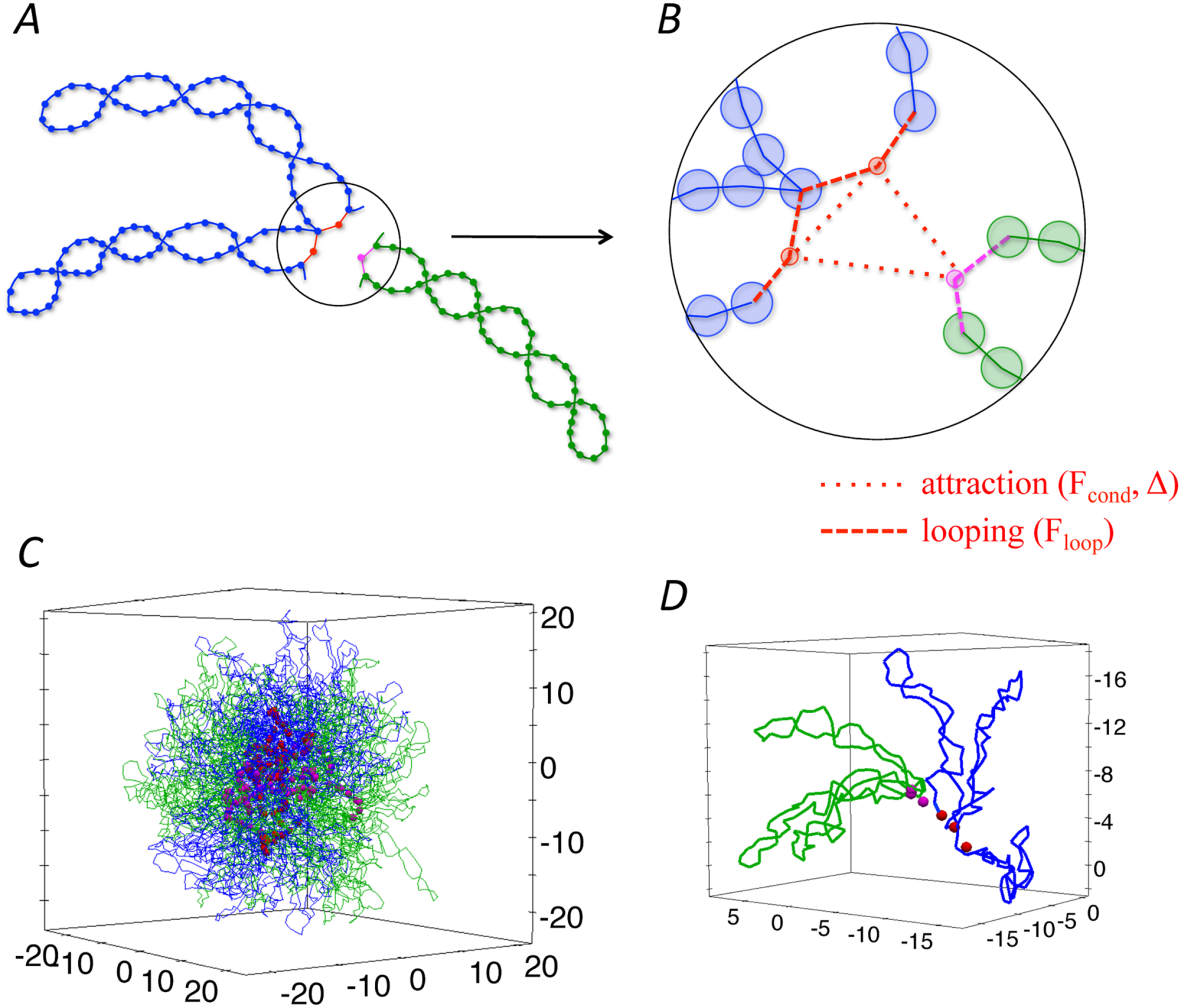
(*A*) Parts of two different chromosome chains (blue and green). (*B*) Enlarged view around the the bases of the loops. Condensins (red and purple points) connect the bases of the loops (dashed lines) and attract each other in cis or in trans (dotted lines). The inter-condensin attraction is controlled by two parameters, *F*_cond_ and Δ, whereas the looping is controlled by *F*_loop_. (*C*) Example of initial configurations, where two chromosome chains (blue and green) are intermingled with each other. (*D*) Part of the initial configuration shown in panel *C*.

We modeled so that each condensin complex has no excluded volume (point particle) and generates two forces: a loop-holding force and an inter-condensin attraction force [5] (Fig 1A and 1B). Note that condensin is a very elongated protein complex whose coiled-coil arms are ∼50-nm long. We consider that its excluded volume is negligible and that the forces can reach the distance of a few of the condensin size. Here, we simplify these forces linearly depending only on the distance between interacting targets and the interacting range [21]. To simulate inter-condensin attractions, we introduced attractive forces among condensin complexes that work in a finite range: the force is negatively proportional to the distance between condensins with factor −*F*_cond_ and is zero when the distance exceeds the threshold distance Δ. The attraction acts among all condensins complexes within the distance of Δ. Employing the loop-holding force, a condensin complex captures two distant monomers on a single chromosome to form and stabilize a loop structure. These monomers become the base-point monomers of the loop. This force is modeled as a harmonic potential with the coefficient *F*_loop_. The loop length was set to be 50 monomers, which corresponds to a few hundred kilobases of DNA in line with available experimental observations [22, 23]. Since neighboring loops share a base-point monomer, consecutive loops are realized.

Considering the fast translocation and looping activities of condensin recently demonstrated in vitro [24, 25], we assume that the loop extrusion process quickly forms chromatin loops in the early prophase stage. The current study focuses on the shaping and segregation processes after the chromatin loops are formed. Thus, we prepare chromosomes with preformed loops as initial configurations (Fig 1C). Moreover, it is possible that chromatin loops have supercoiled configurations within mitotic chromosomes [26]; in fact, it has been shown that condensin I has an ability to introduce supercoils into circular DNA in vitro [27]. The supercoiled configuration promotes the compaction of individual chromatin loops, and is supposed to affect chromosome shaping and segregation. We therefore introduce crossing structures into our chromatin loops to mimic the supercoiled configuration for the initial condition, where each loop has five crossings and its both ends (base points) are connected with each other by the loop-holding force of condensins (Fig 1A). We confirmed that the crossing structures and resulting compaction effects are maintained throughout our simulations, and found that supercoiling of the loops remarkably enhances the segregation speed of chromosomes, see Supporting information (S1 Appendix).

Thus, in our model, condensin functions are controlled by three parameters: the attraction strength *F*_cond_, threshold distance Δ, and loop-holding force strength *F*_loop_. The force parameters *F*_cond_ and *F*_loop_ are normalized by the cut-off energy of the excluded volume interaction. The distance parameter Δ is normalized by the chromatin monomer size *σ*, which corresponds to the size of a condensin complex (See Methods for precise definitions of potentials).

### Simulation methods

**Fig 2:**
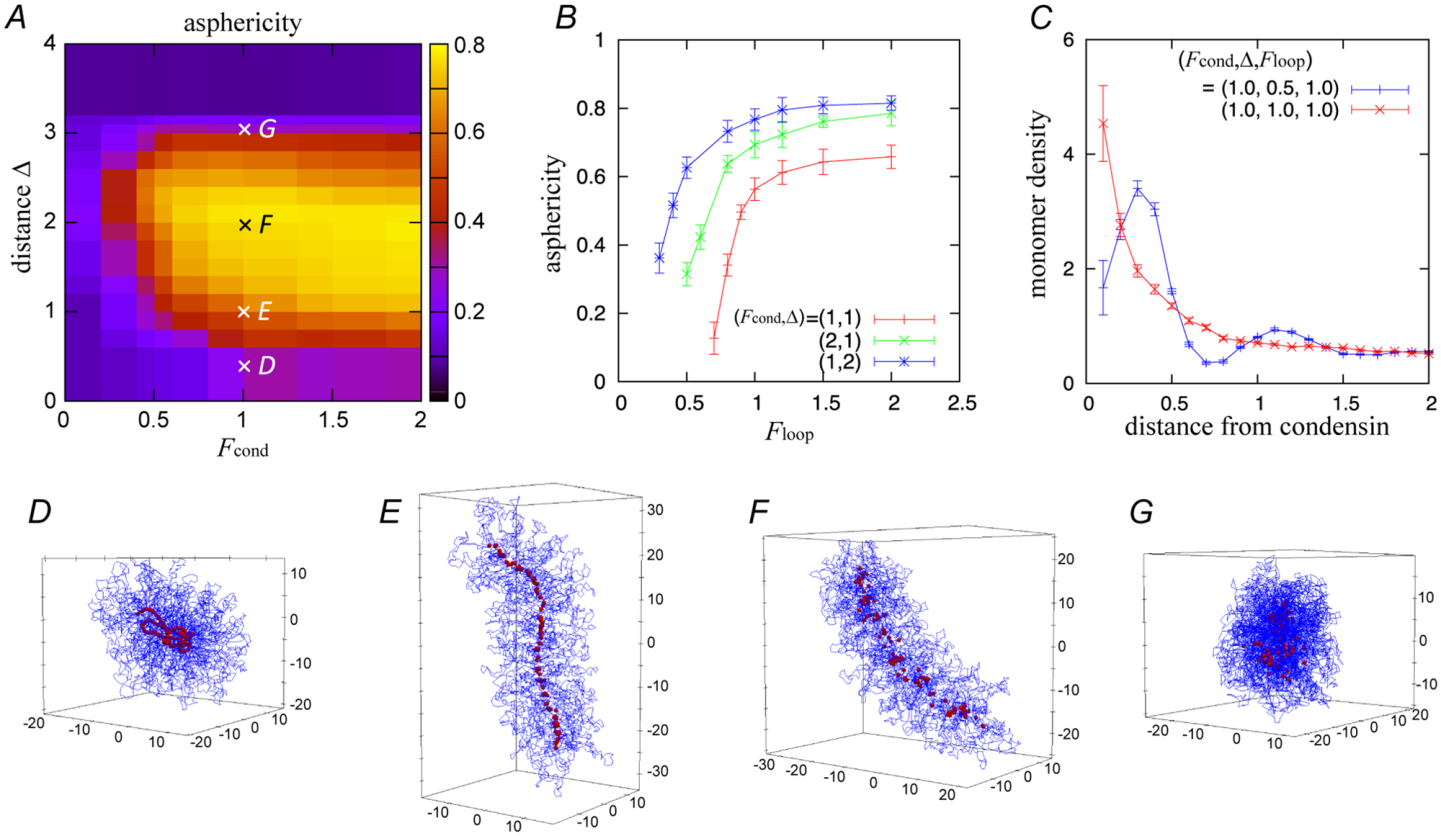
(*A*) Asphericity as a function of the strength *F*_cond_ and the threshold distance Δ of inter-condensin attractions, with $F_{\text{loop}}=1.0$. (*B*) Asphericity as a function of the loop-holding force *F*_loop_ under three pairs of different parameters of the attractions. (*C*) Chromosome monomer density as a function of the distance from condensin at the points *D* and *E* shown in panel *A*. (*D–G*) Example of the configurations observed at the end of the simulations at each point of *D–G* shown in panel *A*. The blue line is the chromosome and the red points are condensins. All simulations are performed employing a single chromosome condition with the number of monomers *N* = 5000 and the number of loops *M* = 100.

**Fig 3:**
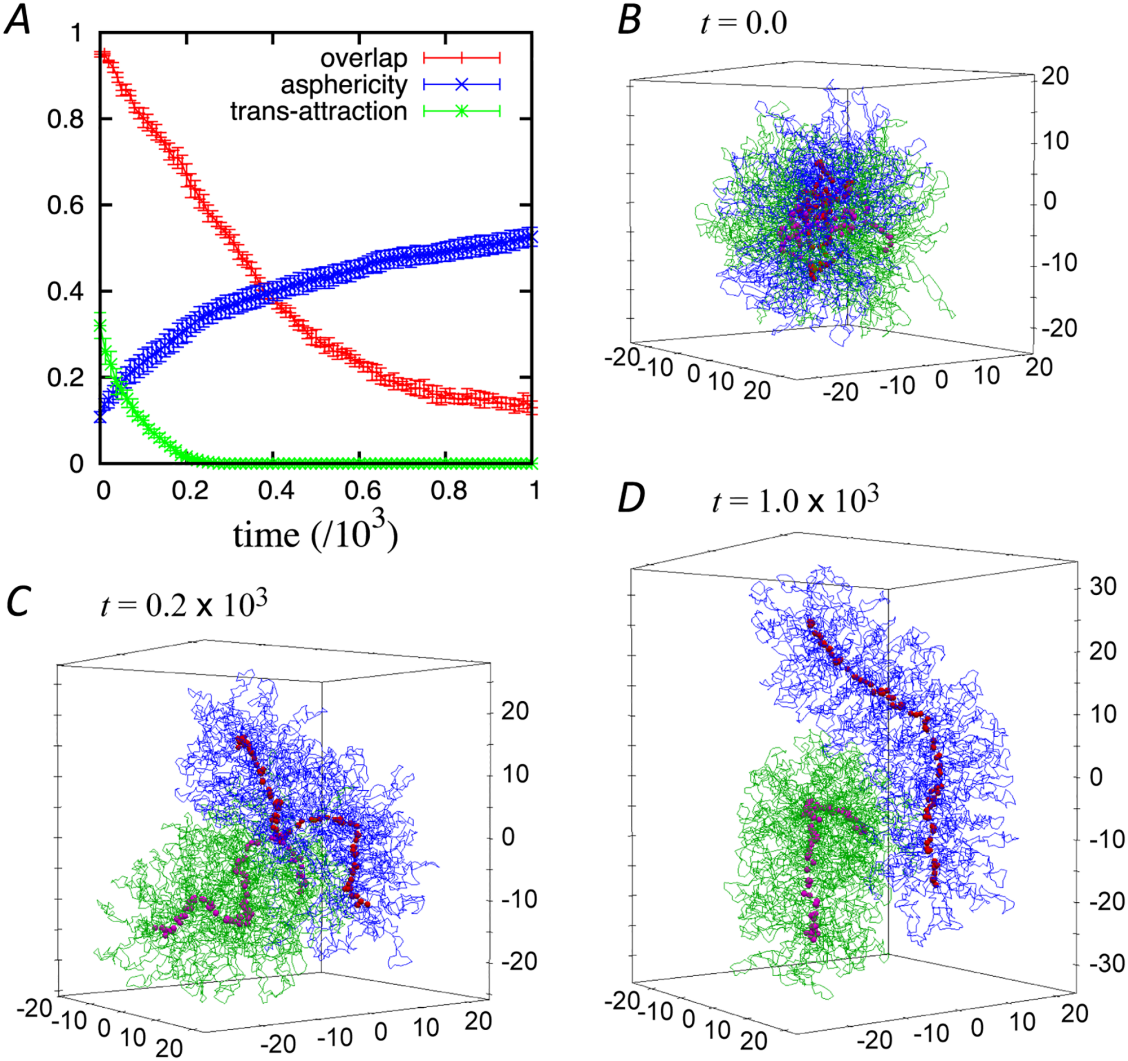
(*A*) Time-course evolution of the asphericity, overlap, and trans-attraction. Configurations of the two chromosomes and distribution of condensins at *t* = 0.0 (*B*), 0.2 (*C*), and 1.0 (*D*). The blue and green lines represent two different chromosomes. The red and purple points are condensins bound to the blue and green chromosomes, respectively. The corresponding dynamics are shown in S1 Movie. Each chromosome has 5000 monomers and 100 loops. (*F*_cond_, Δ, *F*_loop_) = (1.0, 1.0, 1.0).

**Fig 4:**
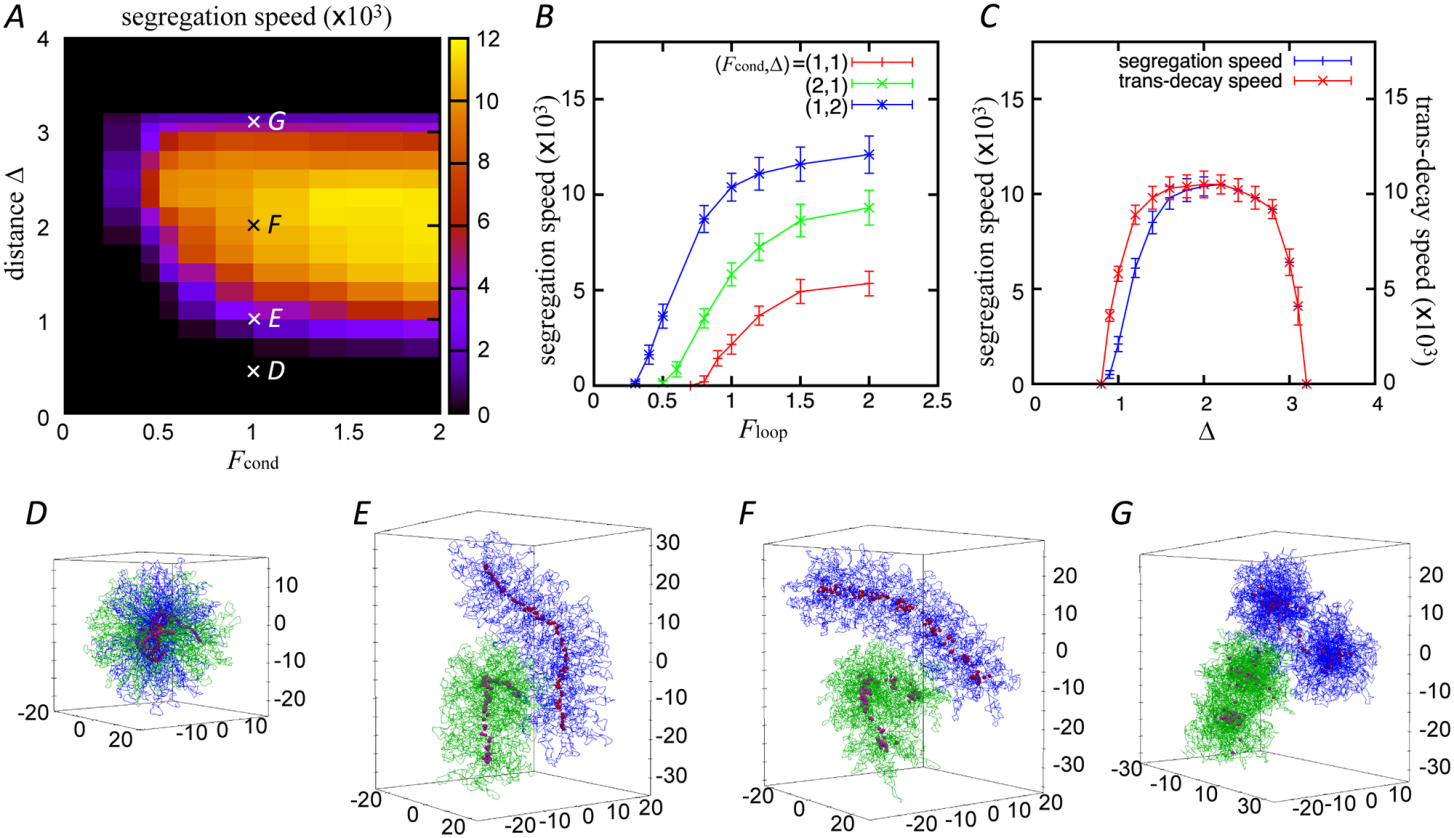
(*A*) Segregation speed as a function of the strength *F*_cond_ and threshold distance Δ of inter-condensin attractions. *F*_loop_ = 1.0. (*B*) Segregation speed as a function of the loop-holding force *F*_loop_ under three pairs of different parameters of inter-condensin attractions. (*C*) Segregation speed and the decay speed of trans-attractions as a function of Δ for *F*_cond_ = *F*_loop_ = 1.0. (*D–G*) Example of configurations observed at the end of the simulations at each point of D–G shown in panel *A*. Each chromosome in all simulations has 5000 monomers and 100 loops.

To set up an initial configuration of one or two chromosomes, we first compacted one(two) chromatin polymer(s) into a spherical shell with diameter 22.85(28.79) to realize the chromatin density of 0.01 in the human nucleus, and equilibrated it. We then simulated consecutive loop structures with crossings in the chromosomes using the loop extrusion process [8, 28] deterministically (Fig. S1). Fig 1C shows the initial configuration of the two chromosomes. As a result of loop formation, the two chromosomes are isotropically compacted and heavily entangled with each other. The initial configuration is insensitive to the model parameters (Table 1). The crossing structures are almost maintained during chromosome shaping and segregation as shown in Figs 2, 3 and 4 (see also S1 Appendix).

**Table 1:** The radius of gyration, *R*_*g*_, asphericity and overlap at initial configuration with each parameter set.

*F*_cond_	Δ	*F*_loop_	*R*_*g*_	asphericity	overlap
1.0	1.0	1.0	22.37	0.16	0.94
1.0	2.0	1.0	21.93	0.22	0.95
0.1	1.0	1.0	22.64	0.19	0.93
2.0	1.0	1.0	22.47	0.14	0.95
1.0	1.0	0.1	25.72	0.17	0.91
1.0	1.0	2.0	21.23	0.15	0.96

We perform simulations using the simulation package ESPResSo [29].

### Condensin functions affect chromosome shaping

Based on the model described above, we first attempted to construct chromosome structures in an equilibrium condition. We define the asphericity as an order parameter to characterize the chromosome shape. Let λ_1,2,3_ be the eigenvalues of the gyration tensor (i.e., the covariance matrix of the configuration of the chromatin monomers, see Methods) with λ_1_ > λ_2_ > λ_3_, so that the normalized asphericity is defined as

$$\begin{array}{c} \text{asphericity}=\frac{\lambda_{1}^{2}-\frac{1}{2}\left(\lambda_{2}^{2}+\lambda_{3}^{2}\right)}{\lambda_{1}^{2}+\lambda_{2}^{2}+\lambda_{3}^{2}}. \end{array}$$ (1)

When the asphericity is small, the chromosome takes on a spherical shape, whereas when the asphericity is large, it displays a rod-like-shape. We observed the asphericity in equilibrium after several-thousand time steps starting from the initial configuration of the chromatin polymer described above.

Fig 2A shows the dependence of the asphericity on the inter-condensin attractions, i.e., the force strength *F*_cond_ and the threshold distance Δ, for *F*_loop_ = 1.0. Fig 2B shows the dependence of the asphericity on the loop-holding force *F*_loop_. The simulations are performed with a single chromosome condition. Our simulations demonstrate that the chromosome shape is strongly affected by both of these predicted activities, inter-condensin attractions and loop stabilization.

For small values of *F*_cond_, Δ, and/or *F*_loop_, the asphericity is also small. Fig 2D shows an example of the configurations observed at (*F*_cond_, Δ, *F*_loop_) = (1.0, 0.5, 1.0). The chromosomes do not change their shapes from the initial configuration and thus remain in a spherical shape. A thin line of condensins, which results from consecutive loop formation, is meandering in this case. With appropriate *F*_cond_ and *F*_loop_, the asphericity displays a unimodal change with Δ: the asphericity monotonically increases with Δ for Δ ≲ 2.5, whereas it decreases with Δ for Δ > 2.5 (Fig 2A). Fig 2D–2G shows examples of the chromosome configurations and condensin distributions with increasing Δ values. As Δ increases, the condensin distributions change from a meandering line to a more straight and rigid structure, which we refer to as the condensin axis. However, the axis shrinks for large Δ values (Fig 2G). Unlike the case of increasing Δ, the asphericity monotonically rises with increasing *F*_cond_ and/or *F*_loop_, as shown in Fig 2A and 2B.

An increase in Δ results in a corresponding increase in the number of attracting condensin pairs, which leads to shrinkage of the line of condensins. Although condensins are here depicted as point particles, they actually take along monomers constituting the base points of loops (Fig 1A). In a lower range, the shrinkage increases the monomer density around the line (Fig 2C). However, its excluded volume repulsion blocks further shrinkage of the line of condensins, thereby producing a stable axis of condensins. The excluded volume repulsion among monomers along with the condensin axis forces result in a uniform monomer density within them, which in turn decreases the meandering of the condensin axis and increases the asphericity, as shown in Fig 2E and 2F. While the loop-holding force gathers the chromatin monomers whose excluded volume interaction causes effective repulsion among condensins, the same force also makes the neighboring condensins in cis to stay in close proximity. Therefore, this force helps to distinguish the neighboring condensins from the others, and also contributes to determining the chromosome shapes in equilibrium. In a high range of Δ > 2.5, the attraction force overtakes these effects of the excluded volume repulsion and loop-holding force. Thus, the condensin line shrinks, leading to the formation of a spherical aggregate (Fig 2G).

### Time-course evolution of chromosome segregation

Next, we investigated the segregation dynamics of two entangled chromosomes (S1 Movie). An initial configuration of two heavily intermingled chromatin polymers was generated as described above, and the segregation dynamics were calculated to observe the time-course evolution of three order parameters: asphericity, overlap, and trans-attraction. The asphericity is defined as above and is expressed as the value for one of the two chromosomes. The overlap is defined by the fraction of monomers within a chromosome that is present in the other chromosome region [20]. The trans-attraction is the fraction of condensin complexes that attract those on the other chromosome (see Methods for precise definitions).

Fig 3A shows the time-course evolution of these order parameters (since there is no comparable time scale, the units are omitted from this analysis). In this simulation, the parameters are set to be (*F*_cond_, Δ, *F*_loop_) = (1.0, 1.0, 1.0), which correspond to those shown in Fig 2E. At the initial stage, the overlap is almost complete (i.e., one) and the asphericity is small (Fig 3B). This indicates that the two chromosomes are heavily entangled with each other and that their shapes are almost spherical. Here, the trans-attraction is almost 0.4, meaning that one-fourth of the condensins attract each other in trans configuration.

As time passes, the extent of overlap and trans-attraction decrease while the asphericity increases monotonically, as shown in Fig 3A. The trans-attraction among condensins goes down more rapidly than the overlap of the chromosomes. Fig 3C shows an example of the configurations when the trans-attraction goes to zero at *t* ≈ 0.2 × 10^3^. Here, the two chromosomes still partially overlap. The condensins start to form a linear axis in each chromosome, but in a meandering manner.

After the trans-attraction reaches zero, the asphericity continues to increase and the overlap continues to decrease in parallel, implying a strong correlation between chromosome shaping and segregation. Eventually, the overlap goes to zero and the asphericity settles down to an equilibrium value. Fig 3D shows the configurations at *t* = 1.0 × 10^3^ when the overlap is ≲ 0.2. The two chromosomes almost completely segregate from each other, and make contact only at small parts of their surfaces. We define the segregation time as the time at which the overlap goes to 0.2, and the segregation speed is calculated as the inverse of the segregation time.

Additionally, we also demonstrated the segregation dynamics involving three entangled chromosomes as shown in S2 Movie.

### Condensin functions regulate chromosome segregation

As shown in Fig 3, the segregation process can be represented by a monotonic decrease in the overlap of the two polymers. Thus, we characterized the segregation speed as the slope of the overlap decrease, and examined the effects of loop stabilization and inter-condensin attractions on the segregation speeds.

Fig 4A shows the dependence of the segregation speed on the two parameters of inter-condensin attractions, i.e., *F*_cond_ and Δ, under the condition where *F*_loop_ is fixed to be 1.0. By contrast, Fig 4B shows the dependence of the segregation speed on the loop-holding force *F*_loop_. Together, these figures demonstrate that the segregation speeds of the entangled chromosomes are strongly affected by both inter-condensin attractions and loop stabilization. On the other hand, the loop length has little effect on the segregation speed (see S3 Appendix).

For small *F*_cond_, Δ, and/or *F*_loop_, the segregation speed is very small. For Δ < 1.0, the entangled chromosomes remain spherical and are entangled for a long time, so that the segregation speed is very low. In this range of Δ, the distance among condensins rarely become shorter than the threshold Δ because of the excluded volume repulsion of the chromatin monomers around condensins (S2 Appendix). Fig 4D shows an example of the configuration observed under this short-range attraction condition: (*F*_cond_, Δ, *F*_loop_) = (1.0, 0.5, 1.0) (also see S3 Movie). The entangled chromosomes stay in the overlapped and entangled states even after a long time of *t* = 10 × 10^3^. The shape of the chromosomes does not change from the initial spherical shape, and the positive axes of the condensins become twisted around each other.

The segregation speed increases when the inter-condensin attraction, *F*_cond_ and Δ (Fig 4A), and/or the loop-holding force *F*_loop_ (Fig 4B) increase(es). Fig 4E shows such an example of configurations with (*F*_cond_, Δ, *F*_loop_) = (1.0, 1.0, 1.0) (see also S1 Movie). The chromosomes take on rod-like shapes and the condensins are localized to the bent axes. In this condition, the chromosomes and condensins are at an equilibrium similar to the configuration shown in Fig 2E. The segregation speed reaches a maximum value at around 2.0 ≲ Δ ≲ 2.5. Fig 4F shows such an example of configurations with (*F*_cond_, Δ, *F*_loop_) = (1.0, 2.0, 1.0) (S4 Movie) after the segregation. In this case, the chromosomes and the condensins axis take on more rigid and straight confirmations, similar to those shown in Fig 2E. The segregation speeds decrease with Δ for Δ > 2.5.

Fig 4C shows the decay speed of trans-attractions among condensins, i.e., the inverse of the time when the trans-attraction goes to zero (Fig 3A), against Δ, where *F*_cond_ = *F*_loop_ = 1.0. For comparison, the segregation speed of chromosomes is plotted under the same condition. For Δ = 1.0, the decay speed of trans-attractions is larger than the segregation speed, as shown in Fig 3A. For Δ < 2.0, the segregation and the trans-decay speeds both increase with Δ, since Δ increases the monomer density and these speeds are enhanced by the monomer repulsion (Fig 4C). For Δ > 2.0, however, the trans-decay speed decreases with Δ since stronger inter-condensin attractions interfere with the trans-decay. The segregation speed also decreases with stronger attractions for Δ > 2.5. Fig 4G shows an example of configurations with a large Δ, (*F*_cond_, Δ, *F*_loop_) = (1.0, 3.0, 1.0) (S5 Movie), where the two chromosomes segregate while maintaining their spherical shapes. When condensins attract each other over a longer range (Δ > 3.2), the two chromosomes fail to segregate completely. Thus, there is an appropriate window for the threshold distance Δ to support the efficient shaping and segregation of mitotic chromosomes.

### Tight correlation between chromosome shaping and segregation

**Fig 5:**
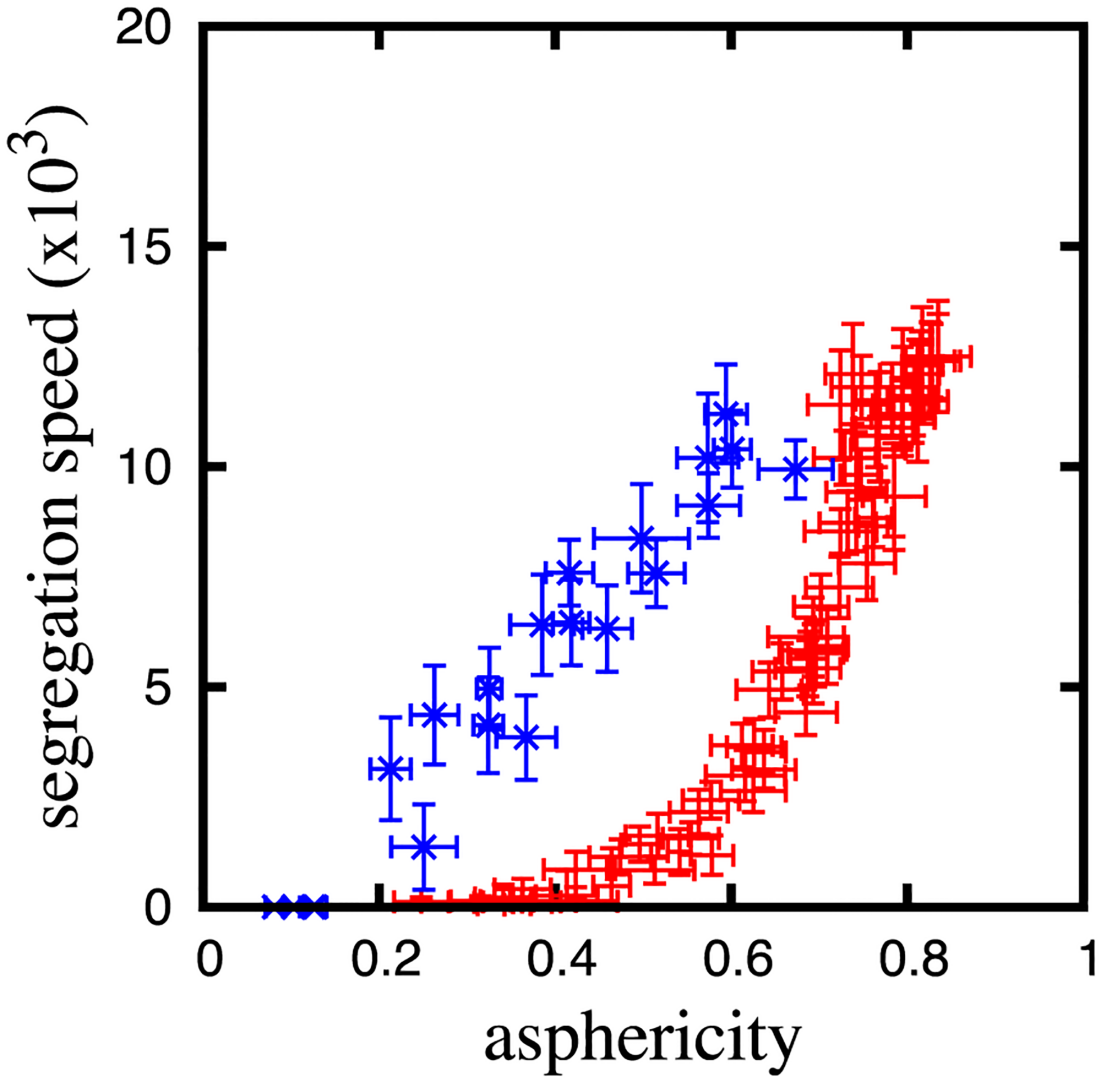
Correlation between the asphericity and segregation speed for various parameter sets of (*F*_cond_, Δ, *F*_loop_). The red plus symbols are the results for Δ < 2.5 and the blue crosses are the results for Δ > 2.5 (S1 Table in supplementary data for sets of detail parameter values).

Comparison of Figs 2A, 2B, 4A and 4B reveals that the chromosome asphericity and segregation speed simultaneously change against the alternation of condensin functions: *F*_cond_, Δ, and *F*_loop_. Fig 5 shows a scatter plot distribution of the asphericity and segregation speed for many parameter sets in the region of 0 ≤ *F*_cond_ ≤ 2.0, 0 ≤ *F*_loop_ ≤ 2.0, and 0 ≤ Δ ≤ 3.5. This plot shows that the asphericity and segregation speed are strongly positively correlated. The correlation exhibits a bifurcation at Δ ≈ 2.5. At Δ < 2.5 (red points in Fig 5), all of the data points are plotted along a single curve even though the parameter conditions are sampled from a three-dimensional space. However, the data from the region of Δ > 2.5 (blue points in Fig 5) configure another branch, where increases in Δ above 2.5 result in considerable decreases of both the asphericity and segregation speed. This result implies that, irrespective of the precise model parameters chosen, chromosome shaping and segregation are likely to be controlled by the same mechanism mediated by condensins.

The bifurcation at Δ ≈ 2.5 is explained as follows. Bellow the bifurcation point, decrease in Δ meanders the condensin axis and entanglement of meandering axes manly inhibits segregation (Fig 4D). Above the bifurcation point, inter-condensin attraction reaches a few diameters of monomers, which brings the attraction among condensins in trans and inhibits the segregation. Therefore, the bifurcation occurs due to the crossover between two inhibition forces against the entropic segregation force.

### Comparison with recent experimental results

**Fig 6:**
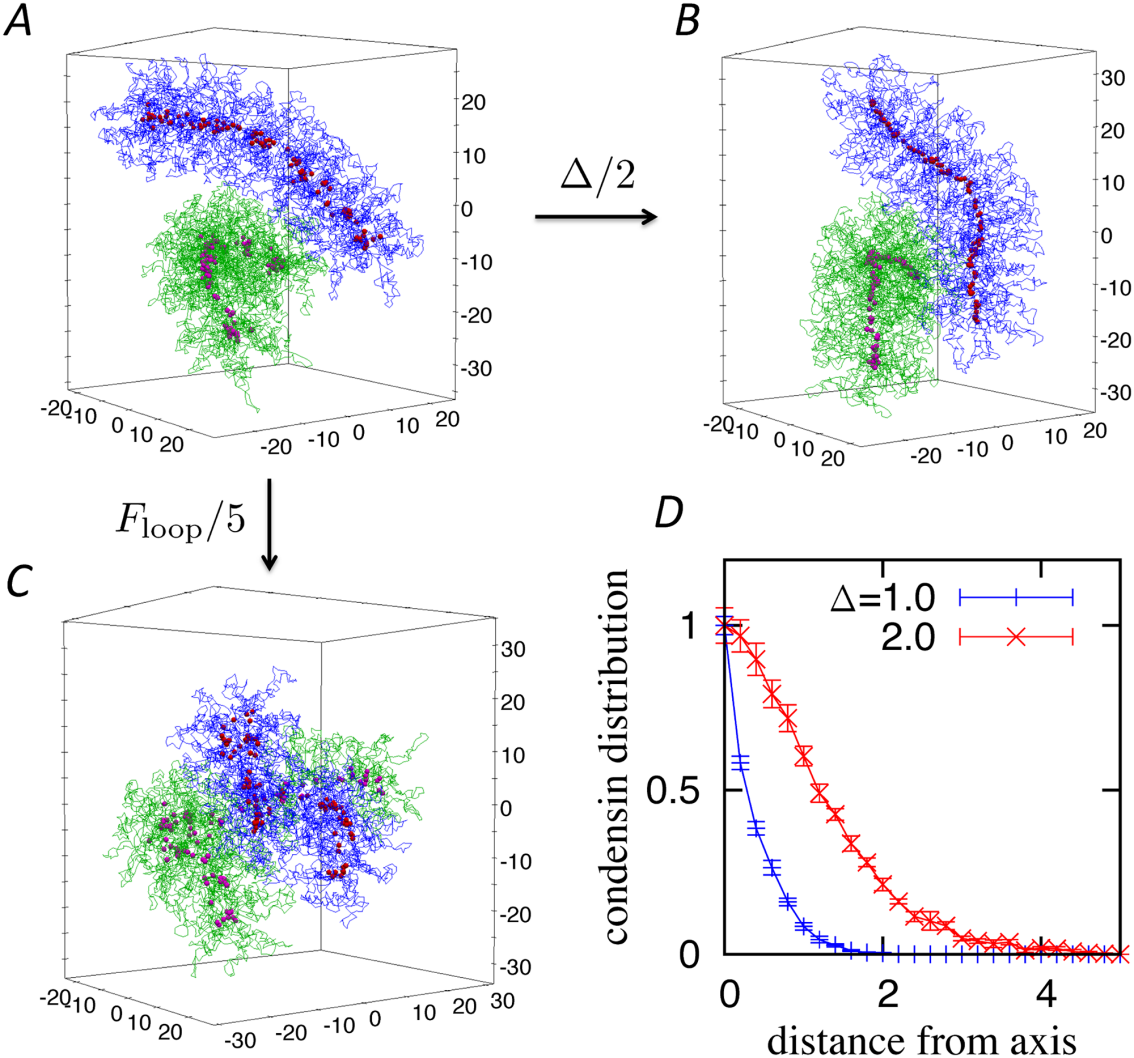
Configuration of chromosomes (blue and green lines) and distribution of condensins (red and purple points) at (*F*_cond_, Δ, *F*_loop_) = (1.0, 2.0, 1.0) (*A*), (1.0, 1.0, 1.0) (*B*), and (1.0, 2.0, 0.2) (*C*). (*D*) Distribution of condensins on the plane perpendicular to the chromosome axis. The distributions are normalized at the origin. The other parameters are fixed to *F*_cond_ = *F*_loop_ = 1.0.

A recent study, reported by some of the authors of the current work, showed that mutant complexes lacking either one of the two HEAT subunits of condensin I, CAP-D2 or -G, produce abnormal chromosomes with highly characteristic defects [16]. In particular, a mutant complex lacking the CAP-G subunit (deltaG tetramer) produced abnormal chromosome structures with very thin condensin axes. In contrast, another mutant complex lacking the CAP-D2 subunit (deltaD2 tetramer) displayed punctate distributions on poorly individualized chromosomes that failed to produce discrete axial structures. Our current simulation results suggest that the defective phenotype of deltaG can be reproduced if the inter-condensin attractions are assumed to occur over a short range, i.e., small Δ. Indeed, as shown in Fig 6B, condensins with a small Δ produce a thinner axis than those with a large Δ. Fig 6D shows the density distribution of the condensins on the plane perpendicular to the axis. As expected, the condensin axis obviously becomes thinner for small Δ values. Thus, when both the loop-holding and inter-condensin attractions work well, rod-shaped chromosomes with thick axes are constructed, as shown in Fig 6A. The phenotype of deltaD2 is more difficult to interpret and reproduce. Intriguingly, however, a similar if not identical structure can be observed when *F*_loop_ is set to be smaller (Fig 6C).

## Discussion

In the current study, we modeled the action of condensins in chromosome shaping and segregation, based on the assumption that they have two molecular activities: chromatin loop formation and inter-condensin attractions [5]. The former function is modeled as the loop-holding force *F*_loop_ and the latter is modeled as the attraction force *F*_cond_ with the threshold distance Δ. We calculated the asphericity and segregation speed as the order parameters for chromosome shaping and segregation, respectively, and show that both strongly depend on the parameters of the presumed condensin activities. It is noteworthy that although both loop formation and inter-condensin attractions occur locally, they can make discrete contributions to the global conformational changes of chromosomes. Our results also demonstrate that the asphericity (i.e., rod-shaping) and segregation speed have a strong positive correlation, implying that the shaping and segregation of mitotic chromosomes might be controlled by a common underlying mechanism. This correlation greatly extends the interpretation of our recent result showing that elongation and compaction increase the segregation speed of entangled polymers [20].

Despite this novel insight, the current study does not address the important issue of how such consecutive loop formation might be achieved. The so-called loop extrusion model suggests one possible mechanism [8, 9]. Goloborodko et al. [28] argued that the loop maturation progresses after initial loop formation via loop extrusion mechanism, possibly mediated by condensins, and that this mechanism may be sufficient for both chromosome shaping and segregation. In the current study, we introduced two parameters of inter-condensin attractions instead of the maturation process, and showed that this postulated activity of condensins also plays a very important role in chromosome shaping and segregation.

Both, the chromosome shape and segregation speed, show the unimodal change with Δ (Figs 2A and 4A). Thus, they appearto be re-entrant phase transitions as observed in DNA condensation by multivalent cations [30]. However, they are qualitatively different phenomena as described below. The aggregation of condensins is observed to monotonically increase with chromosome shape change, which suggest that chromosome shape change is not a re-entrant phase transition. On the other hand, segregation speed demonstrates a unimodal change due to he crossover of the two inhibition forces against the segregation, as described above. This transition can be regarded as a re-entrant phase transition between the entangled and segregated states of two chromosomes.

Since free energy depends on entropy of the chromosome configuration, it is a challenge to calculate the free energy directly. However, DNA condensation has been long investigated in the field of polymer physics, where mean-filed free energy theories has been developed for describing transitions such as the coil-globule transition [31]. The chromosome segregation observed in our simulations and the shape of the phase diagram (Fig 4A) can be explained by the extension of these theories.

Another mechanism for DNA condensation via DNA-bridging proteins was proposed based on computer simulations [32, 33], where DNA elasticity promoted cooperative bindings among these proteins. This mechanism works efficiently in systems with length scales comparable to the persistence length of DNA (50nm) which is similar to the size of the monomer in our model.

In principle, inter-condensin attractions occur either in cis (on the same chromosome) or in trans (between two different chromosomes). Importantly, our simulation demonstrates that trans-attractions observed at an initial time point vanish quickly and are completely replaced by cis-attractions, thereby helping to promote the segregation of the two chromosomes. Our approach also proves to be very powerful given that we could reproduce a highly diverse set of chromosome structures simply by varying the parameters. For instance, the threshold distance Δ modulates not only the asphericity (Fig 2) and segregation speed (Fig 4) but also the width of the condensin axes (Fig 6), providing a potential explanation for the defective phenotype produced by a mutant form of condensin I reported in a previous study [16]. Thus, continued collaborations between theoretical and experimental approaches will be very useful for further dissecting the mechanism of action of condensins and their contributions to mitotic chromosome assembly. Finally, it should be noted that the current model does not distinguish between condensins I and II or consider their differential actions during the process of mitotic chromosome assembly [5]. Therefore, it will be of great interest to take these issues into consideration and to build an advanced form of the model in the future.

## Methods

### Model potentials

As briefly described in the main text, we employed coarse-grained molecular dynamics (MD) simulations with the Langevin thermostat. Specifically, we employed a velocity-Verlet MD integrator with a fixed time step of 0.01. In our MD simulations, we modeled chromosomes as chains consisting of spherical monomers and linearly connecting springs, and modeled condensins as point particles.

Each chromosome consists of *N* monomers with diameter *σ* = 1, mass *m* = 1, and friction *γ* = 1. The potential for chromosomes is described as

$$\begin{array}{c} U_{\text{chrom}}=U_{\text{excl}}+U_{\text{spr}} \end{array}$$ (2)

where *U*_excl_ and *U*_spr_ represent the volume exclusion among monomers and spring interactions between neighboring monomers in the chain, respectively.

The excluded volume interaction *U*_excl_ is described by a Weeks-Chandler-Andersen (WCA) potential, which corresponds to the repulsive part of the Lennard-Jones potential:

$$\begin{array}{c} U_{\text{excl}}=4\epsilon \sum_{i>j\geq 1}^{N}\left[{\left(\frac{\sigma }{r_{i,j}}\right)}^{12}-{\left(\frac{\sigma }{r_{i,j}}\right)}^{6}+\frac{1}{4}\right], \end{array}$$ (3)

for $r_{i,j}<\sqrt[{6}]{2}\sigma$ and 0 elsewhere, where *r*_*i*, *j*_ denotes the distance between the centers of the *i*-th and *j*-th monomers. At *r*_*i*, *j*_ = *σ*, the interaction energy is *ϵ* = 1*k*_*B*_*T*, where *k*_*B*_ and *T* are the Boltzmann constant and the temperature, respectively. To avoid numerical instability, we introduce a cut-off at a maximum energy of the potential *ϵ*_cut_ = 1000*k_B_T*.

The spring interaction *U*_spr_ between neighboring monomers in a chain is described by the harmonic potential:

$$\begin{array}{c} U_{\text{spr}}=\epsilon_{\text{spr}}\sum_{i<N}\frac{1}{2}{\left(r_{i,i+1}-d_{B}\right)}^{2}, \end{array}$$ (4)

where *r*_*i*, *i*+1_ is the distance between the *i*-th and (*i* + 1)-th monomer centers, *d*_*B*_ is the natural length of the springs, and *ϵ*_spr_ is the spring coefficient. We chose the parameters *d*_*B*_ = *σ* and *ϵ*_spr_ = *ϵ*_cut_. The spring has no excluded volume (phantom spring). Thus, spring-spring and spring-monomer can pass through each other, which is mediated by the strand-passage activity of topoisomerase II. Note that actual frequency of the strand passage was low due to the excluded volume of the monomers connected by springs (see S1 Appendix).

The potential for condensins is described as

$$\begin{array}{c} U_{\text{cond}}=U_{\text{loop}}+U_{\text{attr}} \end{array}$$ (5)

where *U*_loop_ and *U*_attr_ represent two functions of the condensins, chromatin loop-holding and inter-condensin attractions, respectively.

With the loop-holding potential *U*_loop_, a condensin interacts with two defined chromatin monomers to make a chromatin loop. The potential is described by the harmonic potential:

$$\begin{array}{c} U_{\text{loop}}=F_{\text{loop}}\sum_{i=1}^{M}\frac{1}{2}({\tilde{r}}_{i,+}^{2}+{\tilde{r}}_{i,-}^{2}), \end{array}$$ (6)

where ${\tilde{r}}_{i,\pm }$ is the distance between the *i*-th condensin and its two interacting monomers, and *M* is the number of condensins that interact with one chromosome by the loop-holding potential; in other words, the chromosome has *M* loops. Since we consider the consecutive loop structures in a chromosome by condensins, the length of the chromatin loop is *L* = *N*/*M*, and the *i*-th condensin bonds to the (*i* − 1)*L*-th and the (*iL* − 1)-th chromatin monomers to make a loop with length *L*, where the order of condensins is aligned with the order of chromatin monomers. *F*_loop_ is the strength of the interaction.

The inter-condensin attraction potential *U*_attr_ is described by the harmonic potential:

$$\begin{array}{c} U_{\text{attr}}=-F_{\text{cond}}\sum_{j<i}^{M^{\prime }}{\left({\bar{r}}_{i,j}-\Delta \right)}^{2}, \end{array}$$ (7)

for ${\bar{r}}_{i,j}<\Delta$ and 0 elsewhere, where ${\bar{r}}_{i,j}$ denotes the distance between the centers of the *i*-th and *j*-th condensins. Δ, *M*′, and *F*_cond_ are the threshold distance, total number of condensins (*M*′ = *M* for one-chromosome simulations and *M*′ = 2*M* for two-chromosome simulations), and the strength of attractions, respectively.

### Initial loop formation process

We established an initial configuration of chromosomes with crossed loops as follows. Consecutive loop structures were made using a loop extrusion mechanism deterministically. The polymer length *N*, loop length *L*, and condensin number *M* have a relation *N* = *LM*. The number of crossing *Cr* determines the structure within a loop.

**Fig 7:**
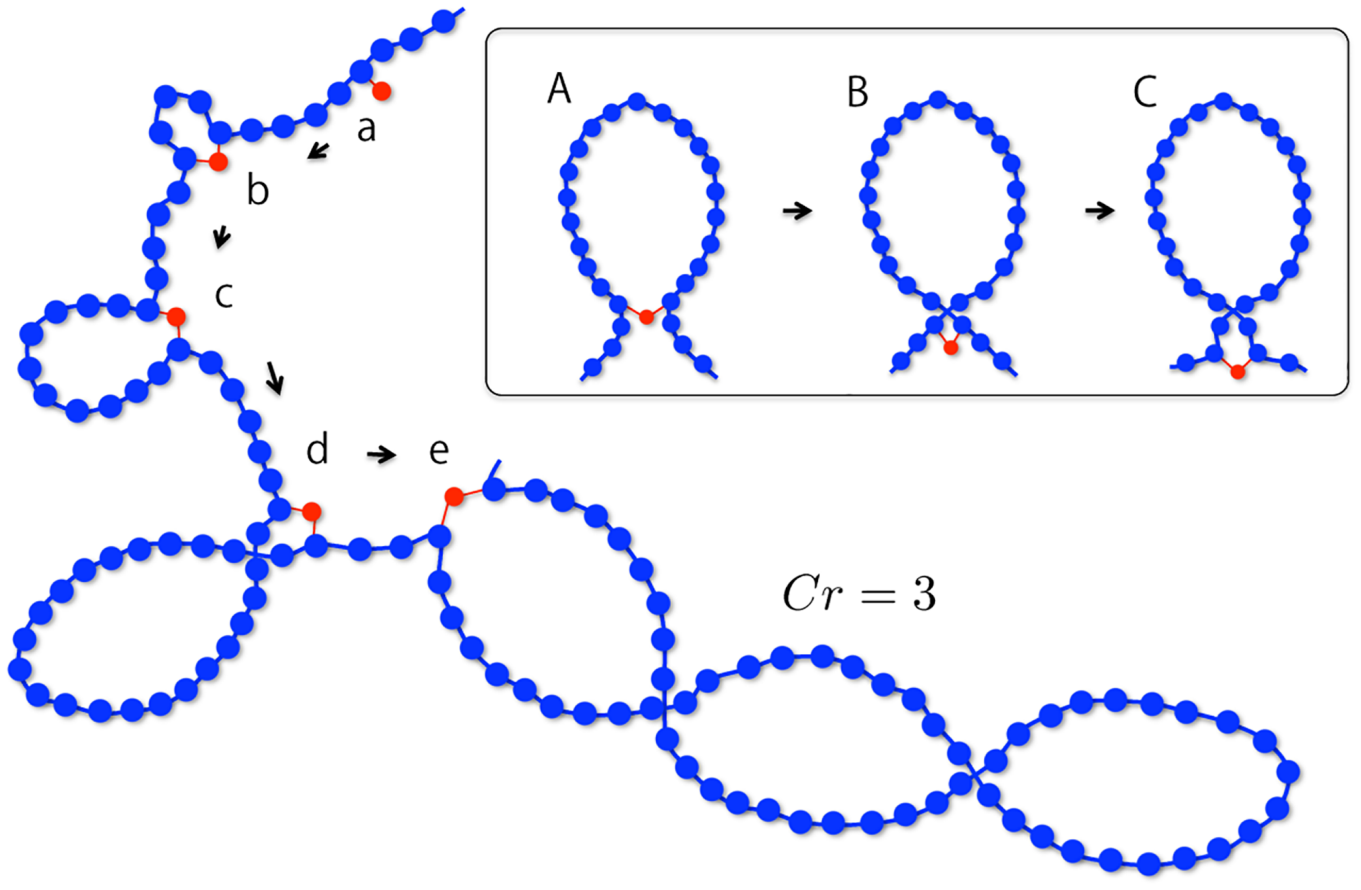
Schematic of the deterministic loop extrusion process. The blue monomers and connecting springs represent a chromosome chain, and the red particles represent individual condensins. The arrows represent the time direction. The inset shows a series of processes that cross in a loop.

Fig 7 shows a schematic picture of the deterministic loop extrusion process with crossings. Each condensin has two bonds. Each bond connects condensin with a chromatin monomer by the harmonic potential. First, the two bonds connect similarly between the *i*-th condensin and the (*i* − 0.5)*L*-th monomer (Fig 7a). The condensins are arranged at regular intervals of *L*. As time passes, the two bonds proceed in a step-by-step manner in the opposite direction along the chromosome chain (Fig 7b). Then, a loop is extruded by each condensin (Fig 7c). After a certain time step, the length of the extruded loop becomes *L*/*Cr*, and then the condensin makes a crossing structure in the loop by changing the spring connection to monomers (Fig 7d). The process of making the crossing structure is shown in the inset of Fig 7. After the length of the extruded loop becomes *L*/*Cr*, the two chromatin springs cross at the same time as the condensin bonds proceed (Fig 7B). Then, the condensin bonds continue to proceed. This loop extrusion process finally results in a loop structure with crossings (Fig 7e).

The initial configuration is insensitive to changing the loop-holding force and inter-condensin attractions. Since each loop is topologically constrained by the crossings, changing *F*_loop_ has little impact on the overall structure of the loops. Moreover, since inter-condensin attractions are set when the condensin distance is less than a certain threshold, changing *F*_cond_ has negligible effects on the initial configuration where different condensin complexes are apart from each other. S1 Table summarizes the radius of gyration, $R_{g}=\sqrt{\sum_{i}\lambda_{i}^{2}}$, the asphericity of one chromosome and the overlap of two chromosome at the initial configuration. These values are almost the same for all the parameter sets.

### Observables

Here, we give the precise definition of the observables. In the main text, the asphericity and the overlap are used as order parameters for chromosome shaping and segregation, respectively.

The asphericity is constructed from the eigenvalues of the gyration tensor (see the main text). The entries of the gyration tensor *G* are given by

$$\begin{array}{c} G_{ab}=\frac{1}{N}\sum_{i=1}^{N}\left({\vec{r}}_{i,a}-{\vec{r}}_{\text{CM},a}\right)\left({\vec{r}}_{i,b}-{\vec{r}}_{\text{CM},b}\right) \end{array}$$ (8)

where *a* and *b* run over the three Cartesian components, and ${\vec{r}}_{\text{CM}}=\frac{1}{N}\sum_{i=1}^{N}{\vec{r}}_{i}$ is the position of the chromosome center of mass. The eigenvalues of *G*, $\lambda_{1}^{2}$, $\lambda_{2}^{2}$, and $\lambda_{3}^{2}$ correspond to the square lengths of the principal axes of the chromosome gyration ellipsoid.

The overlap of two chromosomes is defined as follows. We first define the region of the *i*-th chromatin loop as a sphere with center ${\vec{r}}_{i}^{L}$ and radius $R_{i}^{L}$, which are defined as the center of the mass of loop-consisting units and the maximum distance between the center and monomers, respectively, given by

$$\begin{array}{c} {\vec{r}}_{i}^{L}=\frac{1}{L}\sum_{j=(i-1)L}^{iL-1}{\vec{r}}_{j},\;R_{i}^{L}=\max(|{\vec{r}}_{j}-{\vec{r}}_{L}\left|\right), \end{array}$$ (9)

The chromosome region is represented as a sequence of the spheres. The overlap is defined by the monomer number in the other chromosome region per the total monomer number. Then, the segregation speed is defined by the inverse time when the overlap goes to 0.2.

The trans-(cis-)attraction is the number of condensins that attract those on the other (same) chromosome, divided by the total number of condensins. Here, the attraction acts among all the condensin pairs when their distance is less than Δ.

We define the condensin distribution on the distance from the chromosome axis in Fig 6D. For 1.0 < Δ < 2.5, the condensin axis appears as shown in Fig 2E and 2F. The axis is approximated by a series of straight-line segments connecting the condensin positions with some interval. For example, when we use the interval 5, the condensin axis is approximated by a series of 10 line segments where the total number of condensins on each chromosome is 50. The *i*-th line connects between the 5(*i* − 1)-th and (5*i* − 1)-th condensin. We define the condensin distance from the axis as the distance from the *i*-th straight line for the condensins among the order. The condensin distribution on the distance is then calculated at equilibrium.

All of the observables were averaged over 5 − 10 independent simulations.

## Supporting information

S1 Appendix Effect of crossing structures. (PDF)

S2 Appendix Inter-condensin attractions. (PDF)

S3 Appendix Dependence of chromosome shape and segregation on loop length. (PDF)

S1 Table Asphericity and segregation time. (PDF)

S1 Movie Two chromosome dynamics for (*F*_cond_, Δ, *F*_loop_) = (1.0, 1.0, 1.0). (MP4)

S2 Movie Three chromosome dynamics for (*F*_cond_, Δ, *F*_loop_) = (1.0, 1.0, 1.0). (AVI)

S3 Movie Two chromosome dynamics for (*F*_cond_, Δ, *F*_loop_) = (1.0, 0.5, 1.0). (MP4)

S4 Movie Two chromosome dynamics for (*F*_cond_, Δ, *F*_loop_) = (1.0, 2.0, 1.0). (MP4)

S5 Movie Two chromosome dynamics for (*F*_cond_, Δ, *F*_loop_) = (1.0, 3.0, 1.0). (MP4)
