# Supplementary material for: Modeling the functions of condensin in chromosome shaping and segregation
Source: PLoS Comput Biol. 2018 Jun 18;14(6):e1006152. doi: 10.1371/journal.pcbi.1006152 (PMC6005465; doi:10.1371/journal.pcbi.1006152)
Supplement: S1 Appendix — (PDF) [file pcbi.1006152.s001.pdf]

S1 Appendix: Effect of crossing structures

Maintenance of crossing structures in chromosomes

It is possible that chromatin loops have supercoiled configurations within mitotic chromosomes. The supercoiled configuration promotes the compaction of individual chromatin loops, and is supposed to affect chromosome shaping and segregation. We therefore introduce crossing structures into our chromatin loops to mimic the supercoiled configuration for the initial condition of our simulations.

In order to elicit the compaction effect on shaping and segregation, the crossing structures must be maintained throughout the simulations. However, the crossing structures can be resolved because of the following reasons. The monomer-connecting springs in our model do not have excluded volume (phantom spring), and hence the strand passage activity among them can resolve the crossing structures. Moreover, our chromatin model does not have torsional resistance; as a result, the loops with crossings can resolve themselves into uncrossed loops via thermal fluctuation after enough time. Then, we checked whether the crossing structures are maintained throughout simulations. For this, we chose one chromosome from a simulation (blue line in Fig. $3D$ in the main text) and counted the number of crossings present at the end of the simulation by the following procedures; the configuration of each loop of the chromosome is projected onto x-y, y-z, and z-x planes, the numbers of crossings in these projections are counted, the minimum number of crossings among them is regarded as the number of crossings in the loop, and the numbers of crossings for all loops in the chromosome are summed up. Then, we found a total of 466 crossings in the chromosome out of the 500 crossings that are introduced into one chromosome configuration at the initial condition. Therefore, we conclude that most crossings are maintained throughout the simulation.

This indicates first that strand passage events among phantom springs do not frequently occur. Since we set the monomer diameter and the natural length of springs to be the same value, the steric effect of monomers considerably reduces the frequency of these events. Second, the resolution of crossing structures by thermal fluctuation also does not occur. In general, it takes long time to resolve an intertwined polymer by thermal fluctuation, and the time scale of such resolution is considered

much longer than that of our simulation.

Effect of crossing structures on chromosome segregation

The number of crossing affects chromosome segregation for small Δ values, as shown in Fig. . Fig. *A* shows the time-course evolution of the overlap and the trans-attraction for the number of crossing $Cr = 5$ and 1, where $F_{\text{cond}} = F_{\text{loop}} = 1.0$ and $\Delta = 1.5$. The overlap decreases more rapidly for $Cr = 5$ than for $Cr = 1$, while the trans-attraction decreases for $Cr = 5$ in the same manner as observed for $Cr = 1$. The number of crossing does not change the local structure around condensins, and does not affect the inter-condensin attractions since the attractions are determined locally around the condensins. Accordingly, the number of crossing does not change the decay speed of trans-attractions.

The number of crossing Cr divides a large loop into Cr pieces of small loops by making kinks so that the monomer density in each loop increases with increasing Cr (Fig. *B*). Figure *C* shows the inter-loop penetration, which is the occupancy of loop centers (equation 8 in the main text) by monomers that are not members of the loops, with $F_{\text{cond}} = F_{\text{loop}} = 1.0$ and $\Delta = 1.5$. For small Cr, each loop has large gaping holes (Fig. *B*), and the loops can easily penetrate each other. Thus, the effective repulsion between the loops becomes weak. By contrast, for large Cr, each loop has small gaps (Fig. *B*), and as the inter-loop penetration decreases the repulsion between the loops is strengthened. Through the repulsion dependence on Cr between loops, crossings promote the segregation speed.

Fig. *D* shows the segregation speed as a function of Δ for $Cr = 1$ and 5. The segregation speed is affected by the number of crossing only for small Δ values. For small Δ values, the segregation speed for $Cr = 5$ is larger than that for $Cr = 1$. The loop repulsion is thus the driving force of the chromosome segregation. When the loops have crossings, the repulsive force is stronger and both the cis- and trans-loops rarely overlap (Fig. *C*). Therefore, the segregation speed increases with an increasing number of crossing for small Δ values.

On the other hand, the segregation speeds are almost identical for large Δ values, where the inter-condensin attraction is sufficiently strong so that the cis-loops overlap. This overlap compensates for the weak repulsion of each loop without crossings. As a result, the segregation speed without crossings becomes almost identical to that with crossings.

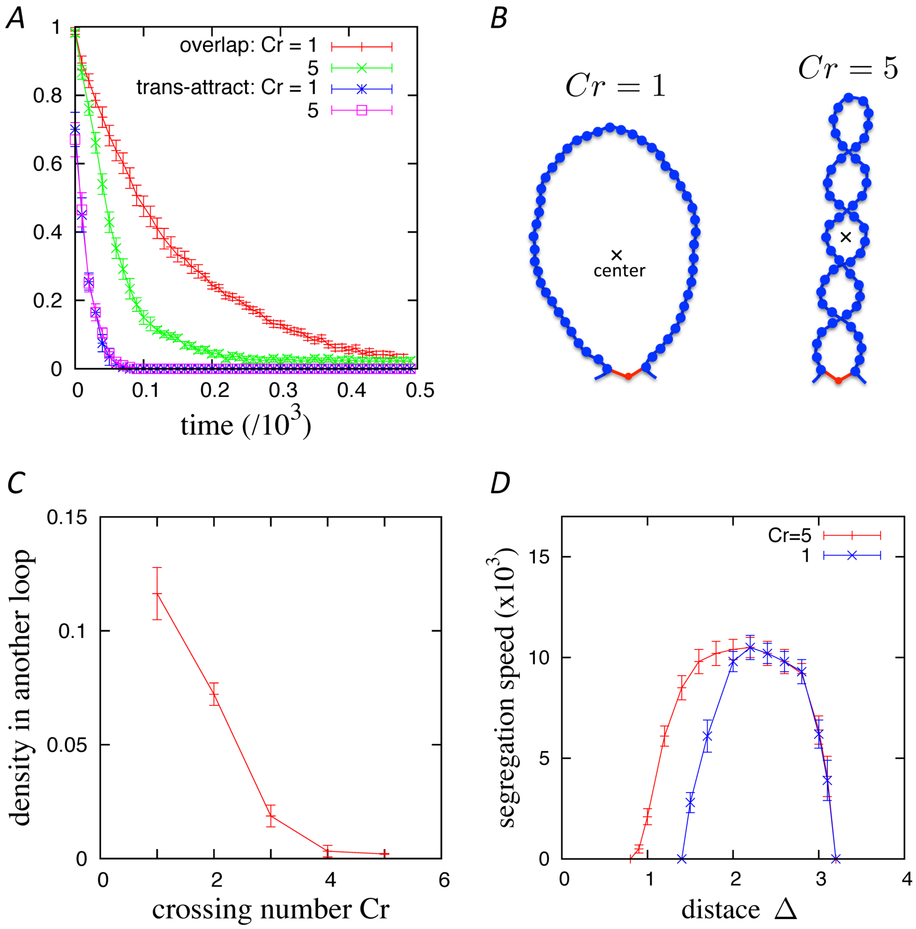

Figure : ***A*** Time-course evolution of the overlap and the trans-attraction for the number of crossing $Cr = 1$ and 5, where $F_{\text{cond}} = F_{\text{loop}} = 1.0$ and $\Delta = 1.5$. ***B*** Schematic of the crossings with $Cr = 1$ and 5. Here, the blue lines represent chromosome loops and the red dots are the condensins. The cross symbols are the loop centers. ***C*** The inter-loop overlap as a function of the number of crossing Cr, where $F_{\text{cond}} = F_{\text{loop}} = 1.0$ and $\Delta = 1.5$. ***D*** Segregation speed as a function of Δ for $Cr = 1$ and 5, where $F_{\text{cond}} = F_{\text{loop}} = 1.0$.
