# Supplementary material for: Modeling the functions of condensin in chromosome shaping and segregation
Source: PLoS Comput Biol. 2018 Jun 18;14(6):e1006152. doi: 10.1371/journal.pcbi.1006152 (PMC6005465; doi:10.1371/journal.pcbi.1006152)
Supplement: S2 Appendix — (PDF) [file pcbi.1006152.s002.pdf]

S2 Appendix: Inter-condensin attractions

Our results showed that inter-condensin attractions are important for chromosome shaping and segregation. Here, we calculate the cis-attraction represented as the mean number of attracting condensins on the same chromosome. Fig. E shows the cis-attraction as a function of Δ at $F_{\text{cond}} = F_{\text{loop}} = 1.0$. For $\Delta < 0.8$, the cis-attraction is almost zero and the condensin complexes do not attract each other at all, so that the chromosomes stay spherical and entangled. For $\Delta > 0.8$, the cis-attraction increases with Δ and condensins attract each other. The cis-attraction increases linearly with Δ for $0.8 < \Delta < 2.5$, which reflects the linearity of the condensin axes on the chromosomes. The cis-attraction increases nonlinearly with Δ for $\Delta > 2.5$, where the condensin axes break into a cluster; further, the chromosomes do not segregate for $\Delta > 3.2$.

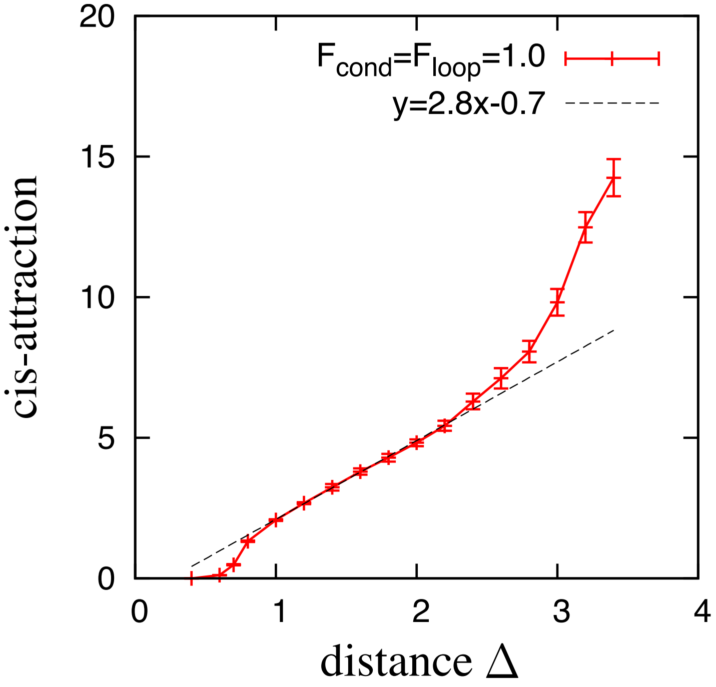

Figure E: The cis-attraction as a function of Δ for $F_{\text{cond}} = F_{\text{loop}} = 1.0$ and $Cr = 5$.
