# Supplementary material for: Modeling the functions of condensin in chromosome shaping and segregation
Source: PLoS Comput Biol. 2018 Jun 18;14(6):e1006152. doi: 10.1371/journal.pcbi.1006152 (PMC6005465; doi:10.1371/journal.pcbi.1006152)
Supplement: S3 Appendix — (PDF) [file pcbi.1006152.s003.pdf]

S3 Appendix: Dependence of chromosome shape and segregation on loop length

In the main text, we investigate the shaping and segregation of chromosomes with fixed loop length (50 monomers). In general, however, loop length can fluctuate in two ways. First, a change in cellular conditions can increase or decrease the binding frequency of condensin complexes, this change may make loop length shorter or longer across whole chromosomes. Second, without any change in cellular conditions, loop length should distribute within one chromosome, and uniform loop length studied in the main text is a simplified model of distributed situations. In this appendix, we examine the dependence of loop length on chromosome shaping and segregation.

First, we examine chromosomes with 40-monomer and 60-monomer loops. In order to minimize the change in the total chromosome size, we provide 5000 monomers for the 40-monomer-loop chromosome and 4980 monomers for the 60-monomer-loop chromosome. Thus, the number of loops, which is the same as the number of chondensin complexes on one chromosome, is 125 and 83, respectively. We also fix the distance among crossings in each loop (introduce the crossing at every five monomers from base point monomers, see Methods for details); a 40-monomer loop has four crossings, and a 60-monomer loop has six crossings. We summarize the numbers in Table S3.1. Asphericity and segregation speed for 40-monomer-loop chromosomes and 60-monomer-loop chromosomes are shown in Fig. S3.1A and B, respectively. $F_{\mathrm{loop}} = F_{\mathrm{cond}} = 1.0$. The black lines indicate data for the 50-monomer-loop chromosomes. Asphericty is measured in a single-chromosome condition and segregation speed is measured under the condition of two entangled chromosomes. Loop length shifts asphericity systematically, which is a direct consequence of the change in the number of condensin complexes (Fig. S3.1A). The unimodal shape of asphericity along with attraction distance Δ appears to be independent of loop length. Segregation speed is also influenced little by loop length (Fig. S3.1A).

Second, we examine chromosomes with non-uniform loop length. We fix the chromosome size ($N = 5000$) and the average of loop length (50), and set loop length distribution to a Poisson distribution (Fig. S3.1C). The distance among crossings in each loop is fixed (a crossing at every 5 monomers from

base point monomers). Then, we generated chromosomes following this distribution, and measured asphericity and segregation speed shown in Fig. S3.1A and B, respectively. The data indicate that the loop length distribution has little effect on both asphericity and segregation speed. In particular, their standard deviations also hardly differ from data for the chromosome with uniform loop length although chromosome shape may fluctuate due to the distribution.

In summary, loop length and its distribution change chromosomal shape regulation and segregation speed only negligibly. In these simulations, we fixed the distance crossings in each loop. S2 Appendix reveals that crossing structures affect segregation speed. Integrating these results, we can conclude that crossing structures are more important for chromosome shaping and segregation than loop length. Crossing structures locally increase monomer density around a loop. We reason that this density regulates shaping and segregation.

loop length	40	50	60
total chromosome size (N)	5000	5000	4980
number of loops (M)<br>(number of condensins)	125	100	83
number of crossing (C_r)	4	5	6

Table S3.1: A summary of structural data on chromosomes with different loop lengths.

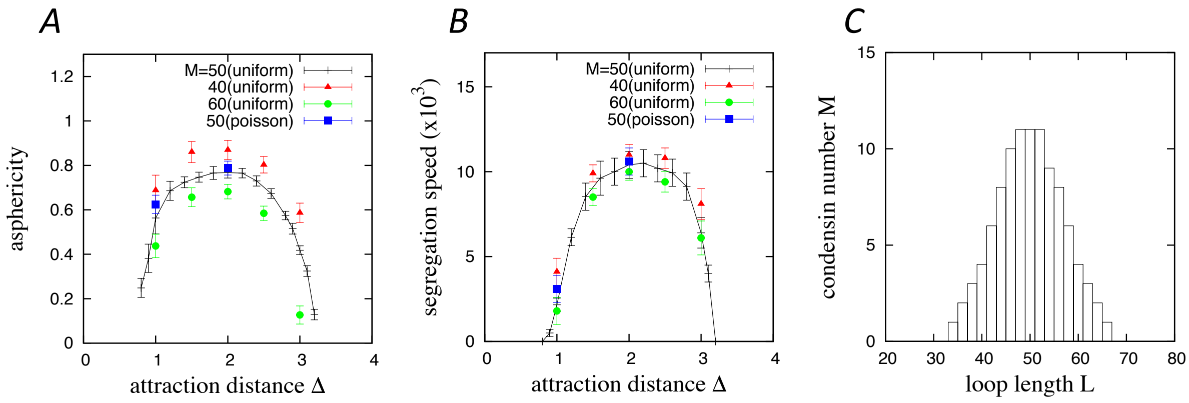

Figure S3.1: ***A*** Asphericity and ***B*** Segregation speed as a function of Δ. $F_{\text{cond}} = F_{\text{loop}} = 1.0$. Data for 40-monomer-loop chromosomes are shown in red, data for 50-monomer-loop chromosomes are indicated in black, and data for 60-monomer-loop chromosomes are shown in green. They have uniform loop length. Data for chromosomes with distributed loop lengths are shown in blue. ***C*** A Poisson distribution of loop length used in simulations. The mean is 50, and the total number of loops is 100.
