# Supplementary material for: Modeling the functions of condensin in chromosome shaping and segregation
Source: PLoS Comput Biol. 2018 Jun 18;14(6):e1006152. doi: 10.1371/journal.pcbi.1006152 (PMC6005465; doi:10.1371/journal.pcbi.1006152)
Supplement: S1 Table — (PDF) [file pcbi.1006152.s004.pdf]

F_{cond}	Δ	F_{loop}	ashpericity	segregation
0.8	1.0	1.0	0.352	0.10
0.9	1.0	1.0	0.474	1.14
1.0	1.0	1.0	0.563	2.16
1.2	1.0	1.0	0.637	3.12
1.5	1.0	1.0	0.685	4.42
2.0	1.0	1.0	0.693	5.83
0.6	1.0	2.0	0.423	0.13
0.7	1.0	2.0	0.513	0.84
0.8	1.0	2.0	0.577	2.43
1.0	1.0	2.0	0.658	5.35
1.2	1.0	2.0	0.702	6.83
1.5	1.0	2.0	0.754	7.81
2.0	1.0	2.0	0.785	9.32
0.3	2.0	1.0	0.342	0.05
0.4	2.0	1.0	0.541	1.25
0.6	2.0	1.0	0.688	5.64
1.0	2.0	1.0	0.767	10.4
1.5	2.0	1.0	0.783	11.5
2.0	2.0	1.0	0.795	11.8
0.2	2.0	2.0	0.554	1.56
0.3	2.0	2.0	0.616	2.98
0.4	2.0	2.0	0.687	5.42
0.8	2.0	2.0	0.794	11.2
1.0	2.0	2.0	0.815	12.1
1.5	2.0	2.0	0.826	12.4
2.0	2.0	2.0	0.835	12.5

F_{cond}	Δ	F_{loop}	asphericity	segregation
1.0	1.0	0.8	0.341	0.21
1.0	1.0	0.9	0.496	1.43
1.0	1.0	1.0	0.563	2.16
1.0	1.0	1.2	0.612	3.67
1.0	1.0	1.5	0.643	5.21
1.0	1.0	2.0	0.658	5.35
1.0	2.0	0.3	0.362	0.12
1.0	2.0	0.4	0.516	1.62
1.0	2.0	0.5	0.626	3.64
1.0	2.0	0.8	0.732	8.73
1.0	2.0	1.0	0.767	10.4
1.0	2.0	1.5	0.808	11.6
1.0	2.0	2.0	0.815	12.1
2.0	1.0	0.5	0.315	0.14
2.0	1.0	0.6	0.423	0.85
2.0	1.0	0.8	0.637	3.52
2.0	1.0	1.0	0.693	5.83
2.0	1.0	1.5	0.761	8.65
2.0	1.0	2.0	0.785	9.32
2.0	2.0	0.2	0.578	1.18
2.0	2.0	0.3	0.625	2.63
2.0	2.0	0.5	0.709	5.92
2.0	2.0	0.8	0.765	10.7
2.0	2.0	1.0	0.795	11.8
2.0	2.0	1.5	0.828	12.4
2.0	2.0	2.0	0.835	12.5

F_{cond}	Δ	F_{loop}	asphericity	segregation
1.0	0.8	1.0	0.248	0.12
1.0	1.0	1.0	0.563	2.16
1.0	1.2	1.0	0.685	6.14
1.0	1.4	1.0	0.724	8.53
1.0	1.6	1.0	0.747	9.82
1.0	2.0	1.0	0.767	10.4
1.0	2.4	1.0	0.729	10.2
1.0	0.8	2.0	0.363	0.41
1.0	1.0	2.0	0.658	5.35
1.0	1.2	2.0	0.762	8.84
1.0	1.4	2.0	0.807	11.2
1.0	2.0	2.0	0.815	12.1
1.0	2.4	2.0	0.748	11.8
2.0	0.8	1.0	0.464	0.47
2.0	1.0	1.0	0.693	5.83
2.0	1.2	1.0	0.742	9.43
2.0	1.4	1.0	0.768	10.9
2.0	2.0	1.0	0.795	11.8
2.0	2.4	1.0	0.724	11.4
2.0	0.6	2.0	0.413	0.26
2.0	0.8	2.0	0.696	5.76
2.0	1.0	2.0	0.785	9.32
2.0	1.2	2.0	0.812	11.5
2.0	1.4	2.0	0.817	12.3
2.0	2.0	2.0	0.835	12.5
2.0	2.4	2.0	0.738	12.1

F_{cond}	Δ	F_{loop}	asphericity	segregation
1.0	2.6	1.0	0.673	9.94
1.0	2.8	1.0	0.574	9.12
1.0	2.9	1.0	0.515	7.58
1.0	3.0	1.0	0.418	6.46
1.0	3.1	1.0	0.324	4.13
1.0	3.2	1.0	0.128	0.00
1.0	2.6	2.0	0.601	10.4
1.0	2.8	2.0	0.459	6.32
1.0	2.9	2.0	0.367	3.85
1.0	3.0	2.0	0.251	1.36
1.0	3.2	2.0	0.103	0.00
2.0	2.6	1.0	0.573	10.2
2.0	2.8	1.0	0.498	8.37
2.0	2.9	1.0	0.385	6.41
2.0	3.0	1.0	0.263	4.36
2.0	3.2	1.0	0.125	0.00
2.0	2.6	2.0	0.594	11.2
2.0	2.8	2.0	0.416	7.59
2.0	2.9	2.0	0.325	4.96
2.0	3.0	2.0	0.213	3.14
2.0	3.2	2.0	0.081	0.00

Table S1: The parameter set, asphericity, and segregation time used for constructing Fig. 5 in the main text.
